# Relation Between the Dantu Blood Group Variant and Bacteremia in Kenyan Children: A Population-Based Case-Control Study

**DOI:** 10.1093/infdis/jiae339

**Published:** 2024-07-09

**Authors:** Silvia N Kariuki, James J Gilchrist, Sophie Uyoga, Alexander Macharia, Johnstone Makale, Julian C Rayner, Thomas N Williams

**Affiliations:** Department of Epidemiology and Demography, Kenya Medical Research Institute–Wellcome Trust Research Programme, Kilifi; Department of Paediatrics; Medical Research Council–Weatherall Institute of Molecular Medicine, University of Oxford; Department of Epidemiology and Demography, Kenya Medical Research Institute–Wellcome Trust Research Programme, Kilifi; Department of Epidemiology and Demography, Kenya Medical Research Institute–Wellcome Trust Research Programme, Kilifi; Department of Epidemiology and Demography, Kenya Medical Research Institute–Wellcome Trust Research Programme, Kilifi; Cambridge Institute for Medical Research, University of Cambridge; Department of Epidemiology and Demography, Kenya Medical Research Institute–Wellcome Trust Research Programme, Kilifi; Institute of Global Health Innovation, Department of Surgery and Cancer, Imperial College London, United Kingdom

**Keywords:** bacteremia, malaria, *Plasmodium falciparum*, Dantu, case-control study

## Abstract

**Background:**

The Dantu blood group variant protects against *Plasmodium falciparum* infections, but its wider consequences have not been previously explored. Here, we investigate the impact of Dantu on susceptibility to bacteremia.

**Methods:**

We conducted a case-control study in children presenting with community-acquired bacteremia to Kilifi County Hospital in Kenya between 1998 and 2010. We used logistic regression to test for associations between the Dantu marker single-nucleotide polymorphism rs186873296 A > G and both all-cause and pathogen-specific bacteremia under an additive model. We used date of admission as a proxy measure of malaria transmission intensity, given known differences in malaria prevalence over the course of the study.

**Results:**

Dantu was associated with protection from all-cause bacteremia (OR, 0.81; *P* = .014), the association being greatest in homozygotes (OR, 0.30; *P* = .013). This protection was shared across the major bacterial pathogens but, notably, was only significant during the era of high malaria transmission pre-2003 (OR, 0.79; *P* = .023).

**Conclusions:**

Consistent with previous studies showing the indirect impact on bacteremia risk of other malaria-associated red cell variants, our study also shows that Dantu is protective against bacteremia via its effect on malaria risk. Dantu does not appear to be under balancing selection through an increased risk of bacterial infections.

## Graphical Abstract

**Graphical Abstract:**
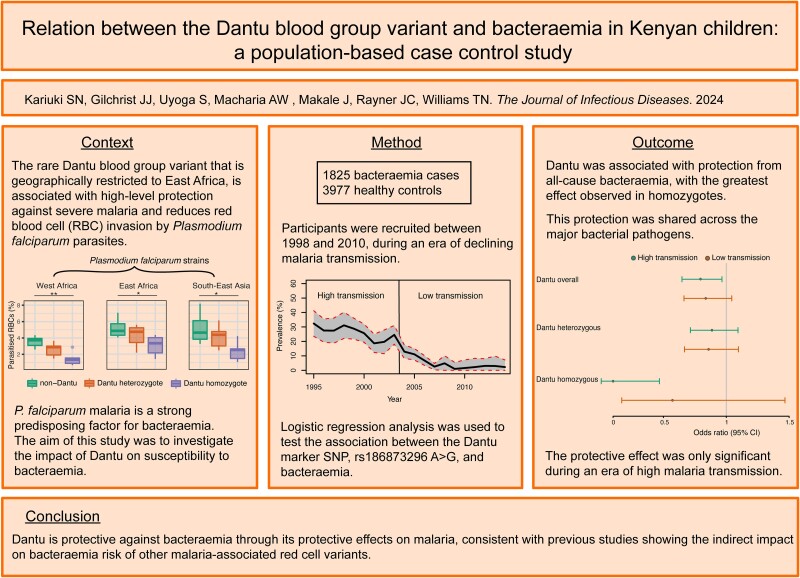

Bacteremia is a major cause of child morbidity and mortality in Africa [1]. *Plasmodium falciparum* malaria is a strong predisposing factor [2–6], contributing to more than half of all bacteremia cases in settings with high malaria transmission [7]. As a consequence, genetic variants that alter susceptibility to malaria infections can also affect the risk of bacteremia in malaria-endemic areas. This is supported by two recent studies conducted on the coast of Kenya. The first found that heterozygotes for the rs334 A > T β^s^ globin mutation (HbAS; sickle cell trait), the most malaria-protective genetic mutation described to date [8–10], are also strongly protected against bacteremia [7], while the second found that G6PD deficiency, which is associated with an increased risk of severe malaria anemia [11], is also associated with an increased risk of bacteremia [12]. Nevertheless, this concept is not universal; for example, subjects who are homozygous for the β^s^ mutation (HbSS; sickle cell disease) who, like those with HbAS, are innately resistant to malaria infections, are at strong risk of bacteremia via other pathophysiological mechanisms [13–20].

Recently, we found that homozygotes for the rare Dantu blood group variant, which is caused by a genetic rearrangement within the glycophorin (*GYP*) gene cluster, are protected against severe and complicated *P. falciparum* malaria infections to a degree that is similar to that afforded by HbAS [21, 22]. Moreover, through a controlled human malaria infection study, we have also shown that Dantu protects against early, nonclinical malaria infections [23]. Nevertheless, despite these strong malaria-protective effects, the distribution of Dantu remains geographically limited. Dantu is most common (minor allele frequency ∼10%) on the coast of Kenya, but it remains rare in all other malaria-endemic populations so far investigated [21, 24]. Possible explanations for this limited distribution are that the causative mutation is recent and has not yet dispersed widely through gene flow, or that the mutation is under balancing selection because it increases the risk of another serious disease.

Dantu appears to protect against malaria because it increases tension in the red cell membrane, which reduces the ability of *P. falciparum* merozoites to successfully invade them [25]. While little is known about the wider health consequences of Dantu, membrane tension has been implicated in the pathophysiology of specific bacterial infections in other contexts [26, 27].

Here, we investigate two competing hypotheses. First, that through its strongly malaria-protective effect, Dantu also protects against bacteremia, and second, that Dantu is under balancing selection via an increased susceptibility to bacteremia. To investigate these two hypotheses, we reanalyzed data from a case-control study of bacteremia, conducted in children on the coast of Kenya during an era of declining malaria transmission.

## METHODS

### Study Population, Design, and Genotyping

The study population has been described in detail previously [12, 28]. In brief, all participants were from Kilifi County on the coast of Kenya. Children aged <13 years who presented to the Kilifi County Hospital with community-acquired bacteremia between 1998 and 2010 were recruited as cases. During this period, malaria transmission declined steadily within the area, potentially due to increasing coverage of insecticide-treated bed nets and improved malaria treatment and control, among other factors [29–31]. By investigating the association between Dantu and bacteremia over time, this therefore provided us with an opportunity to deconvolute any direct impact of Dantu on the risk of bacteremia from any indirect impacts that might result from its malaria-protective effects. The cases were originally recruited into a two-stage genome-wide association study of all-cause bacteremia susceptibility, which was part of the Wellcome Trust Case-Control Consortium 2 (WTCCC2), as described in detail previously [28]. Healthy community controls were selected from among children aged 3–12 months who were resident in the same geographic area as cases, who were recruited to the Kilifi Genetic Cohort Study between 2006 and 2008. Controls were closely matched to cases with regard to sex, ethnic group, and geographic area of residence but not to age.

Following genomic DNA extraction, amplification, and quality control, 1536 bacteremia cases and 2677 healthy controls were analyzed in the discovery phase on Affymetrix single-nucleotide polymorphism (SNP) chip 6.0 arrays at 787 861 autosomal SNPs. In the replication phase, 434 bacteremia cases and 1336 controls were subsequently analyzed on the Illumina ImmunoChip array at 143 100 SNPs. Whole-genome imputation was performed with SHAPEIT [32] and IMPUTE2 [33] using the 1000 Genomes Phase 3 data as a reference panel. Genotypes at the Dantu blood group marker SNP, rs186873296, were well-imputed, with imputation info metrics of 0.98 in the discovery samples and 0.46 in the replication samples.

**Figure 1.**
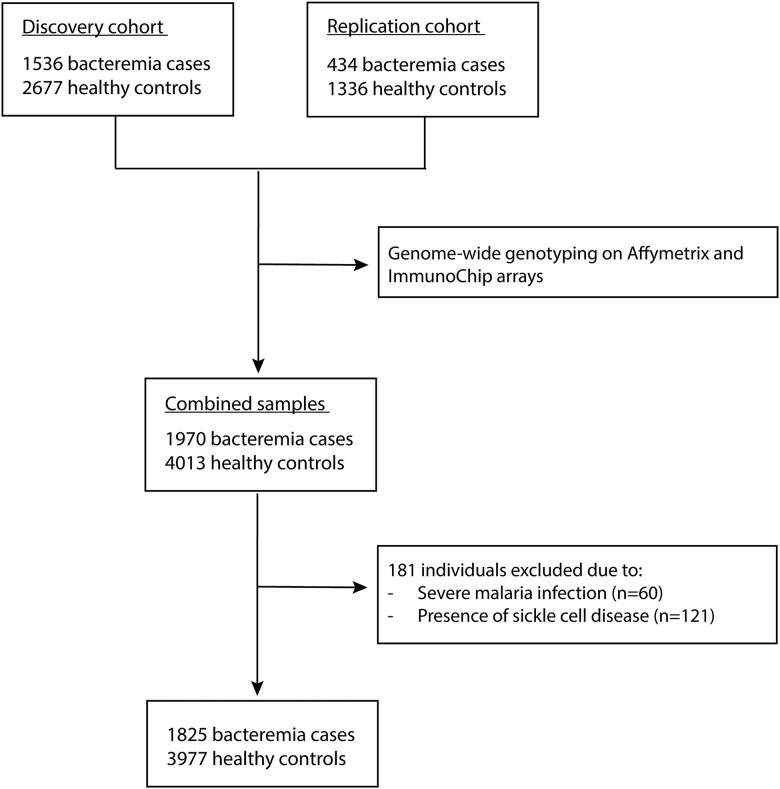
Study design and participant recruitment.

Because the current study was a candidate gene rather than a genome-wide study, we combined the discovery and replication sample in a single analysis, including genotyping platform as a covariate. After removal of cases who also had severe malaria (n = 60) and individuals with sickle cell disease (n = 121), data from 1825 of the 1970 total cases of all-cause bacteremia and 3977 of the 4013 healthy controls were included in the current analysis (Figure 1).

Ethical approval for the study was obtained from the Kenya Medical Research Institute (KEMRI) Scientific and Ethics Review Unit and the Oxford Tropical Research Ethics Committee. Following an explanation of the study, written informed consent was obtained from the parent or guardian of each child included in the study.

### Statistical Analysis

We used logistic regression to test for an association between all-cause bacteremia and the Dantu marker SNP rs186873296 A > G under an additive model. We also estimated the effects of Dantu on bacteremia risk during a period of declining malaria transmission. Based on previous work, the malaria transmission periods were stratified into two phases, a high transmission era between 1998 and 2003 and a low transmission era between 2003 and 2010 [7]. We repeated our case-control analysis for each period using the era-specific cases and the same set of controls. To detect any pathogen-specific effects, we used multinomial logistic regression, comparing 8 case strata among the discovery samples (*Acinetobacter*, n = 153; group A *Streptococcus* spp, n = 169; *Escherichia coli*, n = 174; *Haemophilus influenzae* type b, n = 130; nontyphoidal *Salmonella* spp, n = 169; *Streptococcus pneumoniae*, n = 459; and *Staphylococcus aureus*, n = 190), and 1 stratum for the remaining organisms (n = 381) with controls as the baseline stratum. In all models we included genotyping platform, sex, and the first 4 principal components of genome-wide genotyping data to account for population substructure (Supplementary Figure 1) as covariates. All statistical analyses were performed using R software version 3.6.2 [34].

### Population Attributable Fraction

The population attributable fraction (PAF) for bacteremia was calculated as PAF = *P* (OR – 1) / *P* (OR – 1) + 1, where *P* was the population frequency of the risk genotype and OR was the odds ratio [7, 12]. We also calculated the PAF for malaria using the same formula.

## RESULTS

### Dantu Homozygosity Is Associated With a Reduced Risk of All-Cause Bacteremia

**Figure 2.**
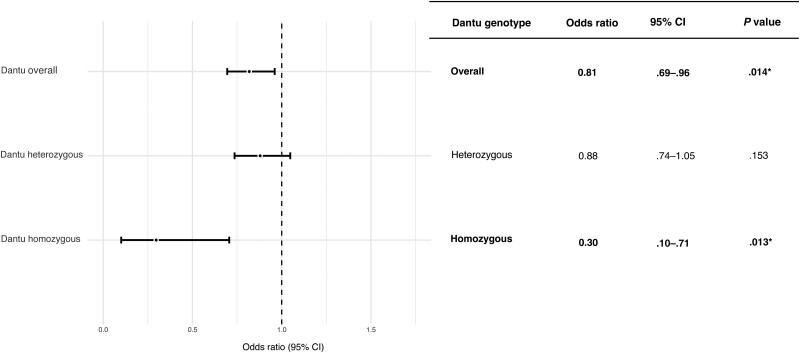
Association between Dantu rs186873296 A > G and all-cause bacteremia. Among the cases, there were 1591 non-Dantu, 229 Dantu heterozygotes, and 5 Dantu homozygotes. Among the controls, there were 3470 non-Dantu, 476 Dantu heterozygotes, and 31 Dantu homozygotes. *P < .05. Abbreviation: CI, confidence interval.

In keeping with previously reported estimates, the minor allele frequency (MAF) of rs186873296:G was 0.07 among the 3977 healthy controls, which included 31 homozygotes. Overall, Dantu was significantly associated with protection from all-cause bacteremia (OR, 0.81; *P* = .014) in our additive model. Strikingly, homozygotes for Dantu had a 70% reduction in bacteremia risk in this population (OR, 0.30; *P* = .013) (Figure 2).

To understand whether the protective effect of Dantu on bacteremia is specific for bacterial infection complicating concurrent malaria infection [35–37] or whether Dantu is protective of bacteremia even without concurrent malaria, we tested the protective effect of Dantu with and without concurrent parasitemia. Overall, Dantu was significantly associated with protection from all-cause bacteremia both with and without concurrent parasitemia (OR, 0.63, *P* = .01 and OR, 0.83, *P* = .03, respectively) (Supplementary Figure 2). This protective effect was statistically significant among Dantu homozygotes without concurrent parasitemia (OR, 0.32; *P* = .02), confirming that the protection afforded by Dantu against all-cause bacteremia is not limited to children with concurrent parasitemia.

### No Evidence for Pathogen-Specific Effects of Dantu

**Figure 3.**
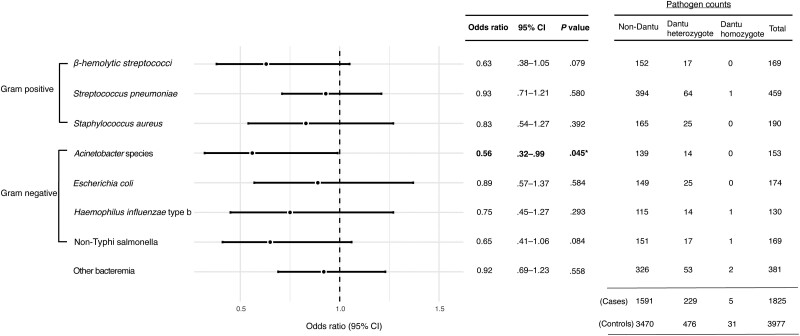
Overall pathogen-specific effects of the Dantu variant. A total of 1825 cases and 3977 controls were included in the analysis (counts of each pathogen type included in the table). *P < .05. Abbreviation: CI, confidence interval.

The Dantu marker SNP, rs186873296:G was associated with a reduced OR across all individual pathogen types, although individually, this protective effect was only significant for *Acinetobacter* spp (OR, 0.56; *P* = .045) (Figure 3). We did not detect genotype-specific associations for specific pathogens among the heterozygotes or the homozygotes (Figure 3, Supplementary Figure 3).

### No Evidence of Protection by Dantu Against All-Cause Bacteremia During an Era of Low Malaria Transmission

**Figure 4.**
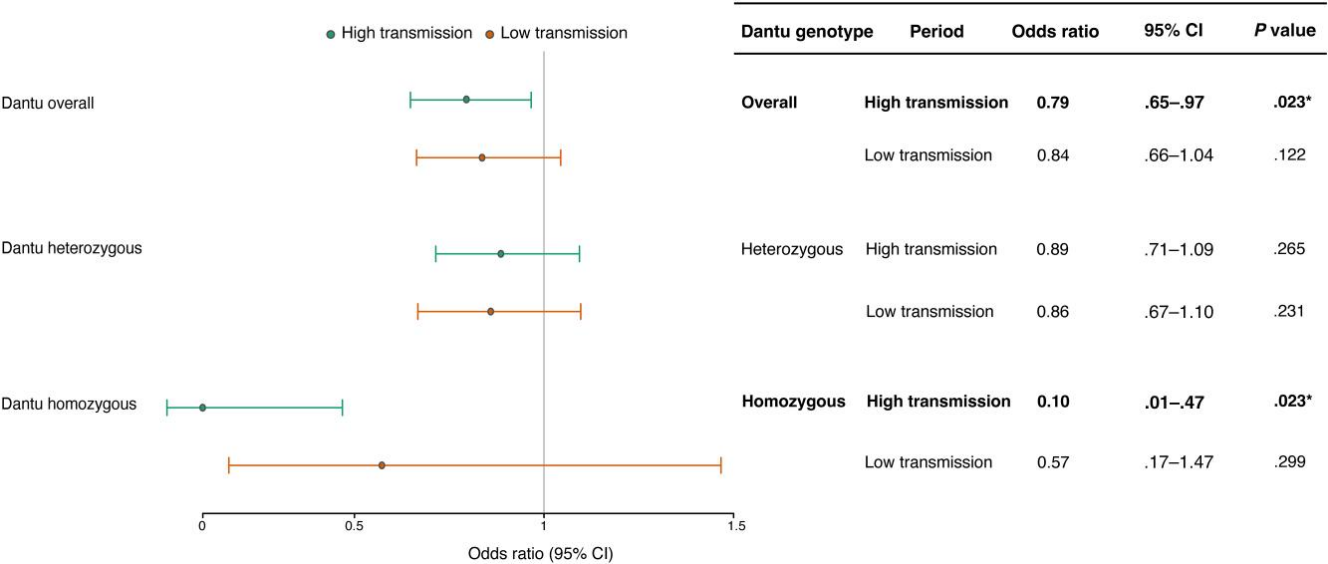
Association between Dantu and bacteremia across malaria transmission periods. Among the cases, there were 728 non-Dantu, 129 Dantu heterozygotes, and 1 Dantu homozygotes during the high transmission period, while in the low transmission period, there were 497 non-Dantu, 86 Dantu heterozygotes, and 4 Dantu homozygotes. Among the controls, there were 3407 non-Dantu, 655 Dantu heterozygotes, and 35 Dantu homozygotes. *P < 0.05. Abbreviation: CI, confidence interval.

As noted above, malaria has been linked to an increased risk of bacteremia. It is therefore important to disaggregate whether the protective effect of Dantu against bacteremia is direct or is indirectly mediated by its protective effect against malaria. Malaria transmission declined significantly in the study region during the period of patient recruitment [7, 29–31], offering us the opportunity to investigate this question further. If the protective association of Dantu against bacteremia is independent of transmission intensities, it would suggest a direct effect, whereas if the effect was reduced as malaria transmission declined, this would be consistent with an indirect effect, mediated by malaria. Our analysis showed that the protective effect of Dantu against all-cause bacteremia was only significant during the high malaria transmission period (pre-2003) (OR, 0.79; *P* = .023), an effect that was greatest in homozygotes (OR, 0.10; *P* = .023) (Figure 4). By contrast, no protective effect of Dantu was seen during the era of low malaria incidence (Figure 4). This suggests that the protective effect of Dantu on bacteremia is most likely mediated indirectly by its protective effect against malaria.

### PAF During the Period of High Malaria Transmission

To understand the overall impact of Dantu on all-cause bacteremia risk at a population level, we calculated the PAF during the high malaria transmission period (1998–2003), during which the OR for the impact of Dantu on bacteremia was 0.79 in our additive model (Figure 4). The PAF for Dantu on bacteremia was 0.02, implying that the presence of Dantu carriers reduced the prevalence of bacteremia within the population as a whole by 2% during the high transmission period. By comparison, given an OR for severe malaria among Dantu carriers of 0.6, and a MAF of approximately 10% [21, 38], the PAF for Dantu on severe malaria was 0.04 among children in this population.

## DISCUSSION

In this case-control study, conducted on the coast of Kenya, we show that the rare blood group variant Dantu is associated with significant protection against all-cause bacteremia. This protection was seen whether or not individuals have concurrent parasitemia, but was only significant during an era of high malaria transmission, suggesting that this protective effect is likely indirect, resulting from the protection that Dantu affords against *P. falciparum* malaria.

Our data indicate a PAF for the protective effect of the Dantu allele against all-cause bacteremia of 2%. As a comparison, given an OR for pneumococcal bacteremia among children with sickle cell trait (HbAS) of 0.36 and a mean HbAS frequency among children in this population of 0.08 [7], the PAF for sickle cell trait (HbAS) on pneumococcal bacteremia is 0.125. In addition, given an OR for severe malaria among children with HbAS of 0.15 and a mean HbAS frequency of 0.08 [38] among children in this population, the PAF for HbAS on severe malaria is 0.312. This larger PAF for HbAS on both pneumococcal bacteremia and severe malaria is mainly driven by the higher frequency and effect size of HbAS in this population, compared to Dantu.

In previous analyses of the same dataset, we have shown that other genes that are associated with malaria risk are also associated with the risk of bacteremia. For example, we have shown that HbAS is associated with significant protection against bacteremia, but that such protection is linked to the prevalence of malaria, indicating that this was mediated by the protective effect of HbAS against malaria [7]. Similarly, we found that G6PD deficiency, a condition that is associated with an increased risk of severe malaria anemia [11, 39], is also associated with an increased risk of pneumococcal bacteremia, but only during an era of high malaria transmission [12]. Our current study accords with these previous observations, supporting the conclusion that Dantu also protects against bacteremia via its protective effect against malaria, underscoring the fact that malaria predisposes to invasive bacterial disease. Several mechanisms have been postulated for how malaria might increase susceptibility to bacterial infections, including through increased hemolysis during malaria infection [2, 40, 41], impaired antibacterial immunity [42–44], and microvascular parasite sequestration in the gut mucosa during malaria infections [45, 46].

One limitation of our study was the small sample size, which limited our power, particularly to explore the pathogen specificity of the association between Dantu and bacteremia. The sample size within each species category was low, and there were even fewer samples to investigate genotype-specific effects for specific pathogens among the heterozygotes and homozygotes. A second limitation of our study is that because Dantu is a complex structural variant that can only be typed directly by sequencing, we used a marker SNP (rs186873296) in our study instead. However, rs186873296 is in such strong linkage disequilibrium with the structural variant in our population (*r*^2^ = 1) that we are confident that it provides accurate data on Dantu genotype in our cohort [22, 24, 38]. This conclusion is supported by previous work in which we showed that the genotyping concordance rate between rs186873296 and the directly typed structural variant was 98% [38]. A third limitation of our study is that we used date of admission as a proxy for force of malaria infection and stratified our analysis by high and low transmission eras based on previous data [7, 12]. As a result, we cannot exclude with confidence the possibility that other confounding factors might also have altered with time during the study period that we have not therefore accounted for in our analysis.

At a global level, the Dantu blood group variant is rare. Although it is found at a MAF of 10% on the coast of Kenya, it is virtually absent in all other malaria-endemic populations studied to date. While Dantu is linked to a haplotype with features of ancient balancing selection [21], the nature of any such balancing effects remains unknown. In this study, we investigated two competing hypotheses: first, that the frequency of Dantu in populations has been held in check by balancing selection through an increased susceptibility to bacteremia, and second, that Dantu is protective against bacteremia through its strong malaria-protective effects. We conclude that the latter hypothesis is correct and that the protective effects of Dantu against both the direct and indirect impacts of malaria are substantial. While the PAF for the protective effect of the Dantu allele against all-cause bacteremia is 2%, it might simultaneously increase the risk for other clinical diseases. While we have not therefore identified the source of any potential balancing effects on Dantu in this study, further studies with larger clinical datasets will be needed to elucidate the wider clinical implications of the Dantu blood group variant.

In summary, in exploring the wider clinical consequences of Dantu, we have found that the Dantu homozygosity confers a significant indirect protective effect against all-cause bacteremia that is secondary to its protective effect against *P. falciparum* malaria. Measures taken to control malaria infection will therefore have broader clinical implications, including indirect protection against invasive bacterial disease in children [47]. The risk of invasive bacterial disease in subjects with Dantu is not acting to balance its malaria-driven selection but contributing to it.

## Supplementary Data

Supplementary materials are available at *The Journal of Infectious Diseases* online (http://jid.oxfordjournals.org/). Supplementary materials consist of data provided by the author that are published to benefit the reader. The posted materials are not copyedited. The contents of all supplementary data are the sole responsibility of the authors. Questions or messages regarding errors should be addressed to the author.

## Supplementary Material

jiae339_Supplementary_Data

## Notes

***Acknowledgments*.** We thank the study participants and their parents for agreeing to participate in this study; the Kenyan Bacteraemia Study Group for participant recruitment and data and sample collection; and the WTCCC2 for allowing us access to genotype data.

***Author contributions.*** S. N. K., J. J. G., and T. N. W. conceived and planned the study. S. U., A. M., and J. M. contributed to sample preparation and phenotypic data compilation. S. N. K. and J. J. G. performed the statistical and computational analysis. J. C. R. and T. N. W. contributed to the interpretation of the results. S. N. K., J. J. G., J. C. R., and T. N. W. wrote the manuscript. All authors read and approved the final manuscript.

***Disclaimer.*** The views expressed in this publication are those of the authors and not necessarily those of the National Institute for Health and Care Research, the Wellcome Trust, the National Health Service, or the UK Department of Health and Social Care. For the purpose of open access, the author has applied a CC BY public copyright licence to any author-accepted manuscript version arising from this submission. This article was published with the permission of the Director of the Kenya Medical Research Institute (KEMRI).

***Financial support*.** This research was funded by the Wellcome Trust (216444/Z/19/Z to S. N. K., 220266/Z/20/Z to J. C. R., 202800/Z/16/Z to T. N. W., and core support 203077/Z/16/Z to the KEMRI–Wellcome Trust Research Programme, Kilifi, Kenya). J. J. G. is funded by a National Institute for Health and Care Research Clinical Lectureship.
