# Supplementary material for: Relation Between the Dantu Blood Group Variant and Bacteremia in Kenyan Children: A Population-Based Case-Control Study
Source: J Infect Dis. 2024 Jul 9;231(1):e10–6. doi: 10.1093/infdis/jiae339 (PMC11793031; doi:10.1093/infdis/jiae339)

**Supplementary Figure 1.** Population structure of study samples. Plots of the first two principal components of genome-wide genotyping data in the discovery and replication cohorts showing stratification of study samples by ethnicity. Individuals are color-coded according to self-reported ethnicity.


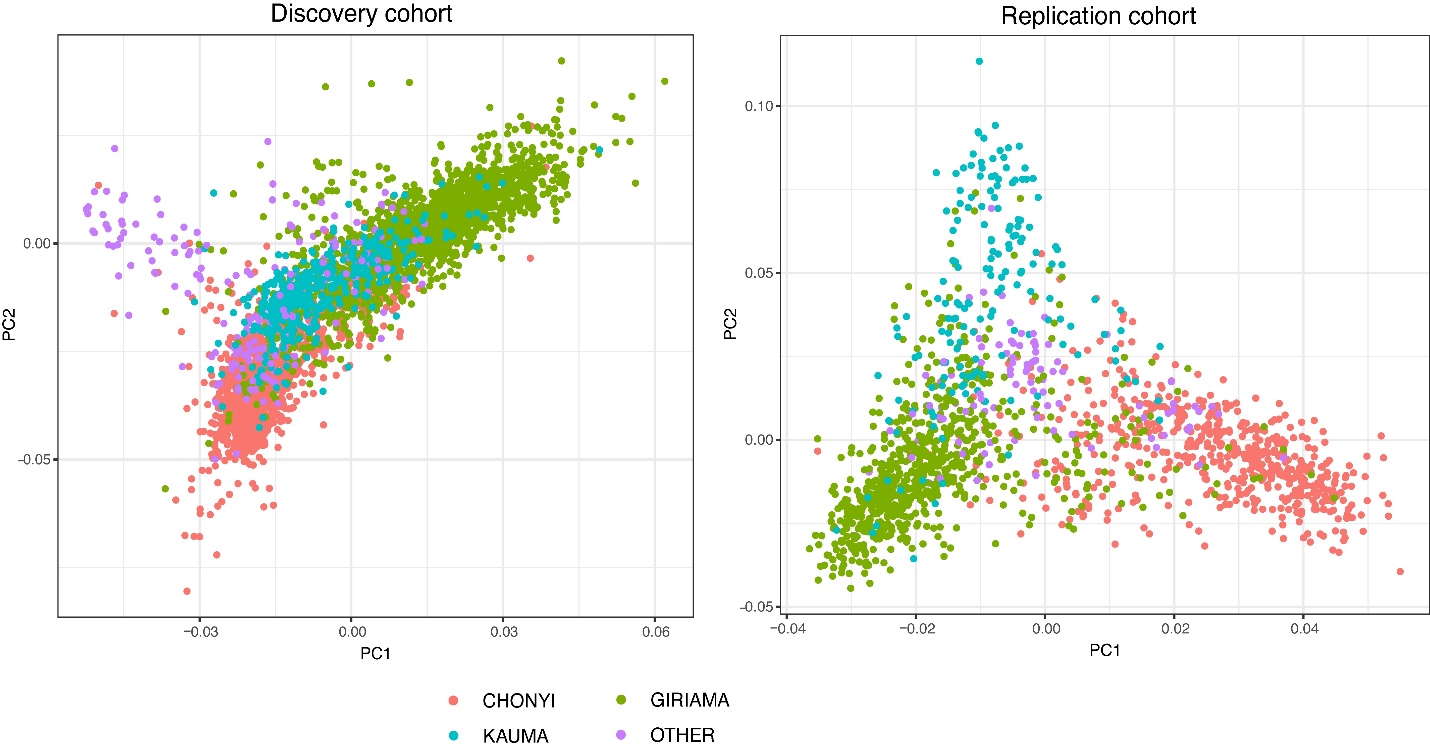


**Supplementary Figure 2.** Association between Dantu rs186873296 A > G and all-cause bacteraemia without concurrent parasitaemia. Among the cases with concurrent parasitaemia, there were 271 non-Dantu, 32 Dantu heterozygotes and 0 Dantu homozygotes, while the cases without concurrent parasitaemia included 1453 non-Dantu, 209 Dantu heterozygotes and 5 Dantu homozygotes.


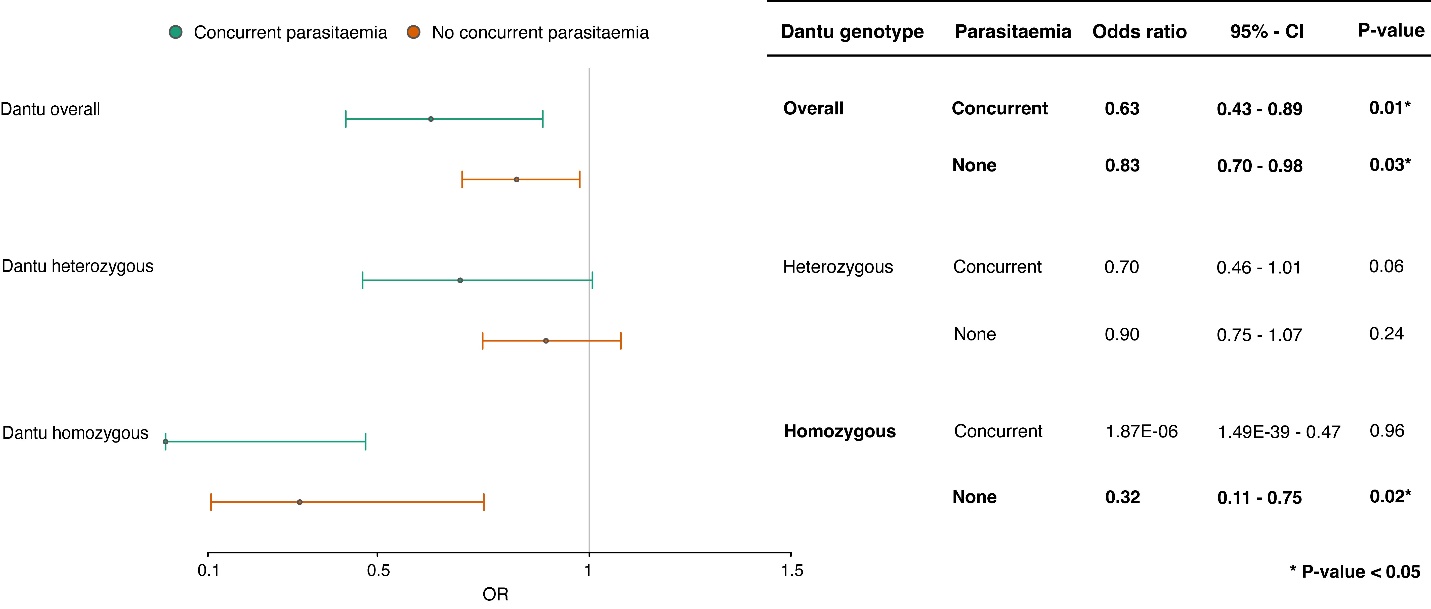


**Supplementary Figure 3**. Pathogen-specific effects of Dantu heterozygous and homozygous individuals.


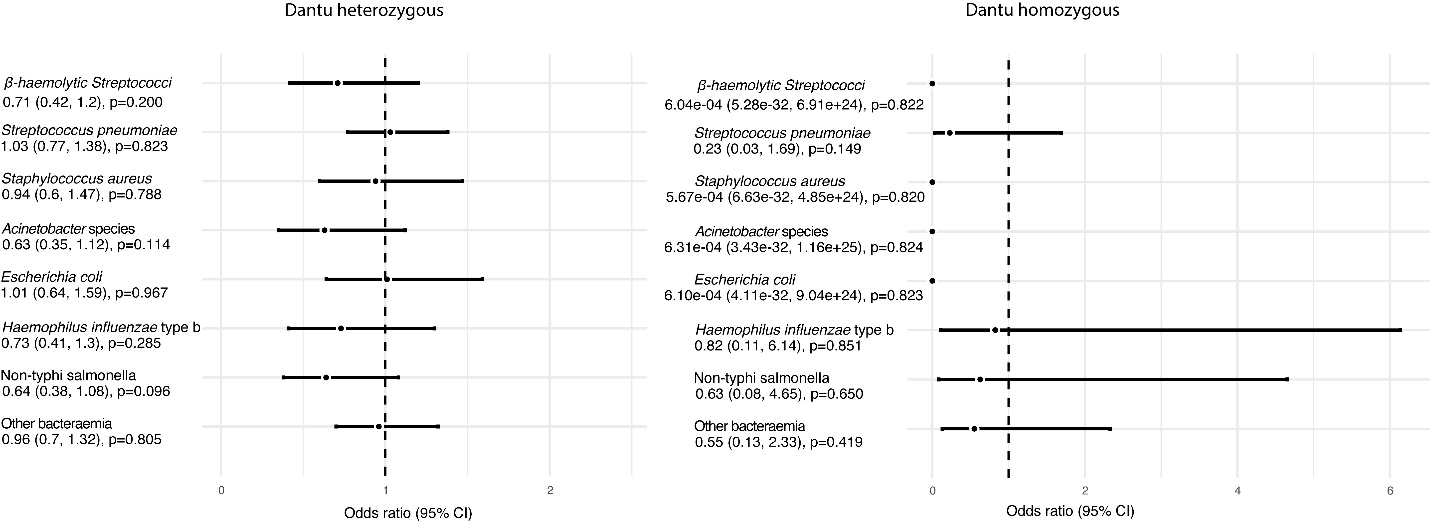

Supplement: jiae339_Supplementary_Data [file jiae339_supplementary_data.zip › jiae339_Supplementary Figures with captions.docx]
