# Supplementary figures and images for: Relation Between the Dantu Blood Group Variant and Bacteremia in Kenyan Children: A Population-Based Case-Control Study
Source: J Infect Dis. 2024 Jul 9;231(1):e10–6. doi: 10.1093/infdis/jiae339 (PMC11793031; doi:10.1093/infdis/jiae339)

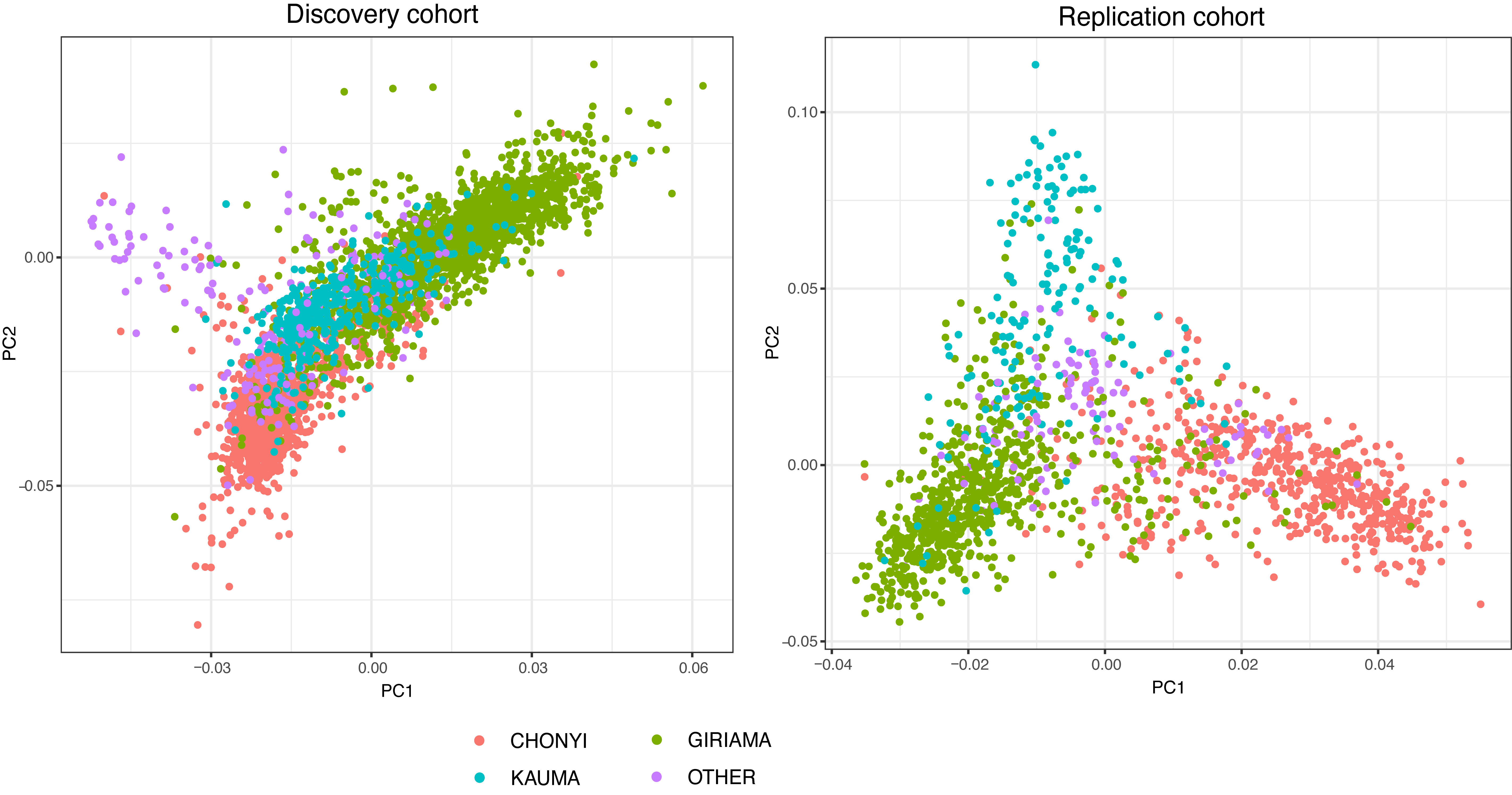

Supplement: jiae339_Supplementary_Data [file jiae339_supplementary_data.zip › Supplementary_Figure_1.png]

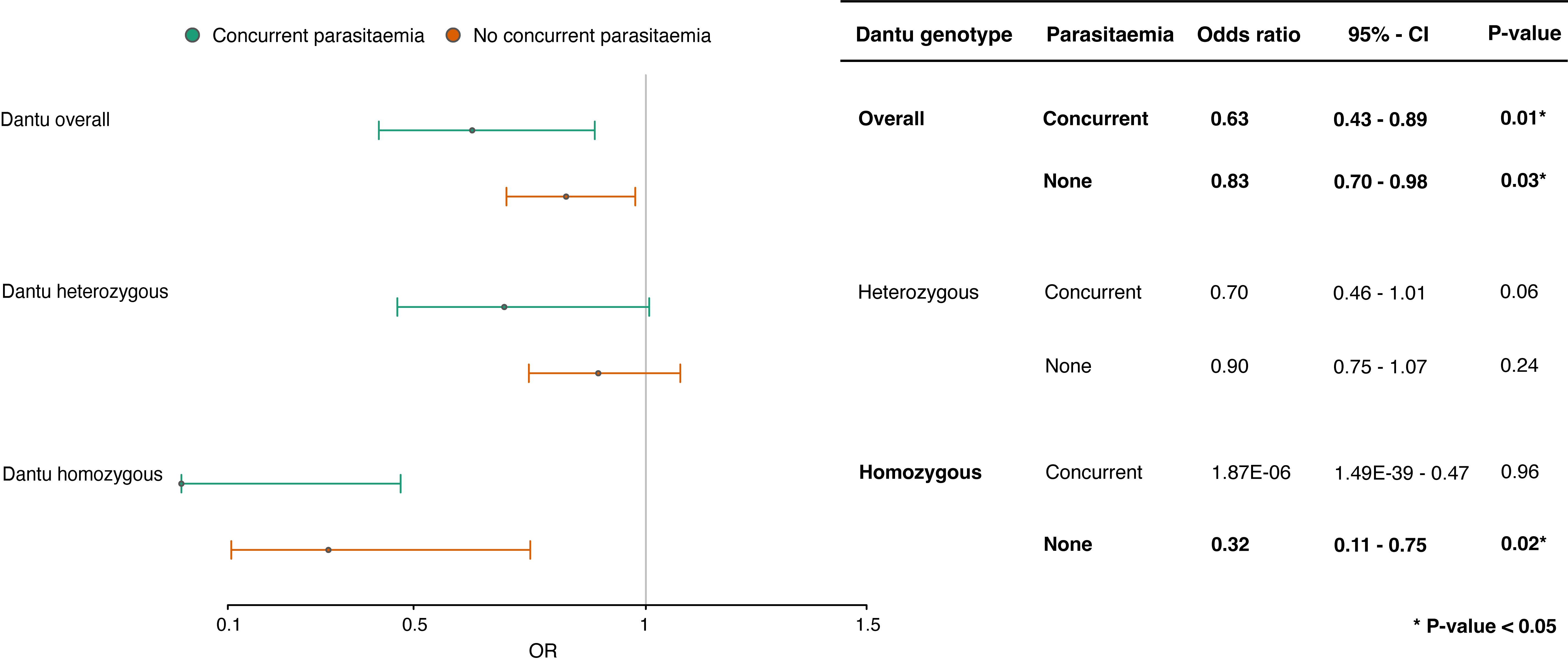

Supplement: jiae339_Supplementary_Data [file jiae339_supplementary_data.zip › Supplementary_Figure_2.png]

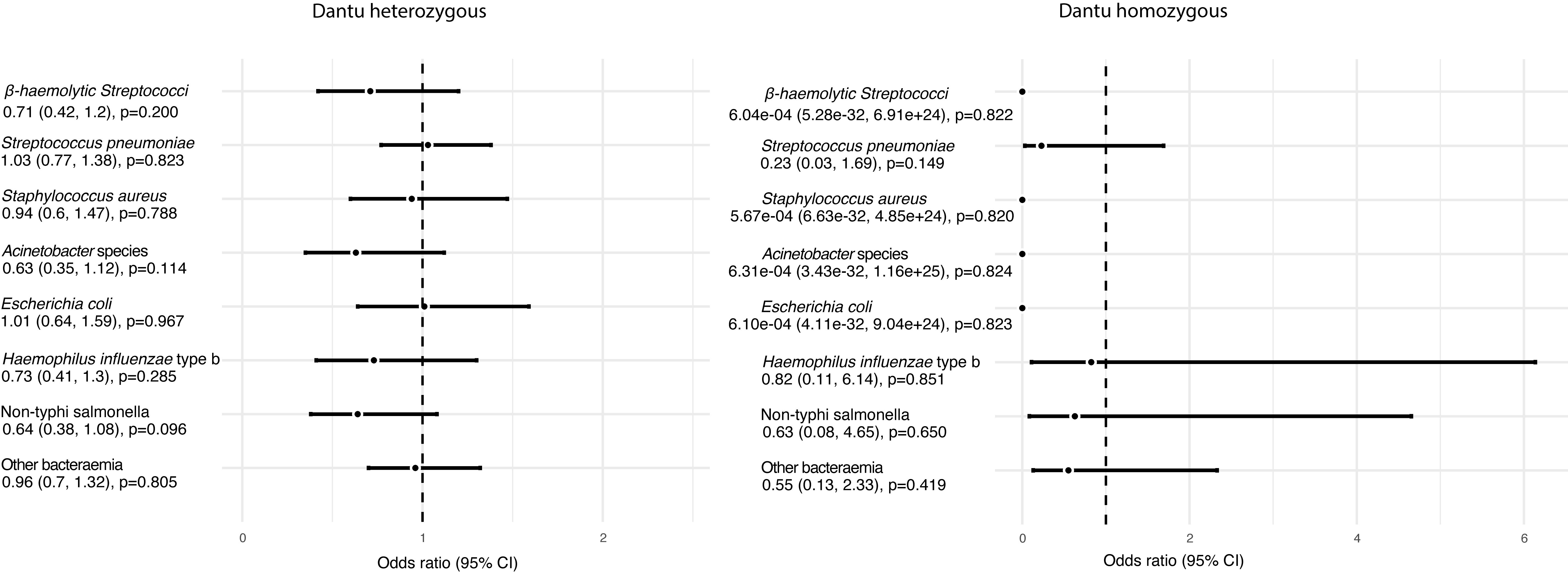

Supplement: jiae339_Supplementary_Data [file jiae339_supplementary_data.zip › Supplementary_Figure_3.png]
